# Development of a Brazilian maize core collection

**DOI:** 10.1590/S1415-47572009005000059

**Published:** 2009-09-01

**Authors:** Ronaldo R. Coimbra, Glauco V. Miranda, Cosme D. Cruz, Derly J. H. Silva, Ramiro A. Vilela

**Affiliations:** Universidade Federal do Tocantins, Centro Universitário de Porto Nacional, Porto Nacional, TOBrazil; 2 Universidade Federal de Viçosa, Viçosa, MGBrazil; 3 Embrapa Milho e Sorgo, Sete Lagoas, MGBrazil

**Keywords:** germplasm collection, *Zea mays*, breeding, landraces, selection

## Abstract

The aim of this study was to evaluate methods for developing a Brazilian maize core collection. For an initial survey of the active collection, passport information, as well as characterization and evaluation of accessions, were taken into consideration, these then being divided according to geographic region and kernel-type. Multiple sampling methods were evaluated. The strategy of constant sampling generated extensive alterations in extract accession frequency. The multivariate strategy with dispersion graphs and principal components associated with the Tocher method was considered efficient for identifying the most divergent genotypes. The multivariate strategy generated greater alterations in the variance of traits. The average number of traits revealed few modifications with the various sampling strategies used. Therefore, the active collection could be considered as possessing a satisfactory amount of information for most of its accessions. Moreover, the multivariate strategy generated modifications in the variance of the traits, independent of sampling intensity.

## Introduction

Germplasm collections were initially created to preserve crop-plant genetic resources (Brown, 1989a). These collections possess a vast number of accessions which present many problems, such as poor organization and handling, a lack of data regarding their characterization and evaluation, and the want of or insufficient passport information, thereby resulting in the inadequate use of genetic resources in breeding programs (Brown, 1989a). Plant genetic variability can be used in association with hybrid performance in maize (Miranda *et al.* , 2003, 2008). The diallel crosses is very used to evaluate the heterosis in different species when the genetic variability is not available (Oliveira *et al.* 1999). The genetic variability is very important to identify resistant genotype for disease (Silva *et al.*, 2003).

A set of strategies is under development for facilitating the use and conservation of germplasm, this including the creation of core collections (Hodgkin *et al.*, 1995; Gepts, 2006). A core collection is made up of a limited sample of accessions, chosen to represent genetic variation within the germplasm collection itself (Frankel and Brown, 1984; Brown, 1989a,1989b). The development of a core collection aids in concentrating efforts on the characterization and evaluation of germplasm, reduces costs and makes resources available for other activities, such as germination tests which facilitate access to the germplasm collection (Brown, 1989b).

Genetic variation among plant populations is not a random occurrence, but takes place in a structured manner according to a series of factors as, for example, geographic origin. Therefore, an important point to be considered in the development of core collections is the application of sampling strategies that identify and partition the maximum variation possible (Brown, 1989a; Frankel and Brown, 1984). With this in mind, various methodologies can be used, based upon both the available data and objectives of the core collection.

Upadhyaya *et al.* (2001, 2002, 2006) developed core collections for chickpea (*Cicer arietinum*), peanut (*Arachis hypogaea* L.) and pigeonpea [*Cajanus cajan* (L.) Millsp.]. The core collection for chickpeas was stratified by country of origin and data on 13 quantitative traits were used as entries forclustering by the Ward method. Various tests, including the comparison of mean data by using the Newman-Keuls test, variance by the Levene test and distribution by the χ^2^ test, besides the Wilcoxon rank-sum non-parametric test for different traits, indicated that genetic variation in these traits in the entire collection was preserved in the core-subset.

The peanut core collection was formed with accessions that were evaluated for morphological, agronomic, and quality traits in the rainy and post-rainy seasons. The Ward clustering method was used to separate core collection accessions into groups by similarity. The Newman Keuls test for means, the Levene test for variance and the χ^2^ test for frequency distribution analysis of different traits, indicated that variation in the core collection was preserved in the mini core-subset. In further research, a pigeonpea core collection with 146 accessions was constituted by evaluating 1290 accessions. Examination of the data for various morphological and agronomic traits indicated that almost all of the genetic variation and most of the co-adapted gene complexes present in the core-subset were preserved in the mini core-subset.

Li *et al.* (2005) formed a Chinese maize core collection. The collection was first divided into landraces and inbred lines. The percentage of the original collection to be included in the core was 7%, based on a previous study of sampling strategies for maize. Each group was sequentially stratified based on administrative provinces or regions and kernel types. A clustering method was applied for further stratification. A logarithmic strategy was used to determine the number of entries in the core at each step. The process resulted in a maize-core comprising 951 landraces and 242 inbred lines. The Shannon-Weaver diversity index and means were used to validate the core. This core collection can be effectively employed in further in-depth research and maize-improvement.

Gutierrez *et al.* (2003) compared two methods for classifying Uruguayan maize landraces; racial classification obtained through visual assessment and numerical classification. The Ward method was used for numerical classification and the Modified Location Model (MLM) to refine the resultant groups. The Ward-MLM strategy generated more homogeneous groups than those formed by a preliminary racial classification. Numerical classification produced groups with clearly distinct traits, in terms of numerical variables, that were superior to those formed on the basis of racial classification alone.

The aim of this study was to evaluate methods for the development of a Brazilian Core Collection from the Maize Germplasm Active Collection of Embrapa Maize & Sorghum.

## Material and Methods

The Maize Germplasm Active Collection of Embrapa Maize & Sorghum - Brazil, is composed of 1,753 accessions. All available information on these accessions was assembled, including passport information, characterization and evaluation. The traits that were previously evaluated in the Active Collection of Embrapa Maize & Sorghum and considered in the present work are: 1) Kernel type (KT): dent, semident, flint and, semiflint; 2) Days to anthesis (DA); 3) Days to silking (DS); 4) Plant height (PH, meters); 5) Ear height (EH, m); 6) Percentage of broken plants (PBP); 7) Percentage of lodging plants (PLP); 8) Leaf number above the superior ear (LNA); 9) Leaf number per plant (LN); 10) Stalk width (SW, cm); 11) Number of ears (NE); 12) Ear length (EL, mm); 13) Ear width (EW, mm); 14) Number of kernel rows (NKR); 15) Number of kernels per row (NKR); 16) Ear weight (EW, g); 17) Grain weight per plant (GW, g); 18) Cob weight (CW, g); and 19) weight of 1000 Kernels (KW, mg).

The accessions were divided according to geographic region and kernel type, in order to identify the number of extracts in the former core collection. The Brazilian geographic regions involved were Sul, Cerrados, Cerrados-Norte, Amazônia, Caatinga and Agreste-Litoral. The kernel types considered were dent, flint, semi-flint and semi-dent.

As to methodology, two sampling intensities were used for obtaining genetically divergent groups, 30% of the accessions according to Yonezawa *et al.* (1995) and 10% according to Brown (1989a). Strategies were according to Li *et al.* (2004), and were as follows:

- Constant (C30 and C10): amount of accessions sampled within each constant extract, and random sampling of the accessions in extracts, with a sampling intensity of 30% or 10%.

- Proportional (P30 and P10): amount of accessions sampled in proportion to the size of the extract and random sampling of the accessions in extracts, with a sampling intensity of 30% or 10%.

- Logarithmic (L30 and L10): amount of accessions sampled in proportion to the logarithms of accession frequency in each extract and random sampling in each extract, with a sampling intensity of 30% or 10%.

- Random (R30 and R10): stratification by geographic origin and kernel type was not considered. Accessions were chosen through the generation of random numbers, with a sampling intensity of 30% or 10%.

- Multivariate (MV30 and MV10): accessions were first grouped in relation to the place of evaluation (Sete Lagoas, MG or Janaúba, MG), and then in relation to geographic region and kernel type. The number of accessions sampled in Janaúba and Sete Lagoas was proportional to the size of the extract. Principal components analysis was carried out, the number of variables diverging in accordance with the availability of information on each extract. Data standardization was carried out for an average of zero and variance of one. The analysis of graphical dispersion was carried out with scores from the first principal components. Thereby, the most divergent accessions within each extract were chosen.

Comparisons between the active collection and developed core-collections were undertaken. Alterations in accession frequency in each extract as a result of sampling averages, trait variance and retention-index variability were taken into consideration. The χ^2^ test was used in checking whether accession frequency in each extract of the core collection remained equal to that in the active core collection. The F-test was used to determine whether trait variance in the extract was equal to that in the active collection. Comparison between averages was carried out by means of the Student t-test. Therefore, the data-set was a sample of the active and not the entire collection. The variability retention index was calculated according to Diwan *et al.* (1995).

## Results

There are 1,753 accessions for landraces gathered from different regions of Brazil in the Maize Active Collection of Embrapa Maize & Sorghum. In the case of certain regions, there is sufficient information for developing core collections. Nevertheless, this is not so as regards of the statesEspírito Santo, Piauí and Tocantins , thus making further additions necessary. The majority of accessions in this active collection are from São Paulo, Bahia, Roraima and Rio Grande do Sul (data not shown).

After organizating the database there was sufficient information with 806 maize-accessions to develop a core collection, as well as to compare different sampling strategies. The active collection was divided into groups in accordance with kernel type and geographic region. Of these 806 accessions, 374 possessed dent kernels, 326 semi-dent, 83 flint and 33 semi-flint. In Sete Lagoas-MG, 608 accessions were evaluated and 198 in Janaúba-MG. The number of accessions by region is as follows: Cerrados 228, Caatinga 205, Amazônia 147, Sul 124, Cerrados-Norte 58 and the Agreste-Litoral 44.

Sampling of 30% and 10% of the landrace accessions resulted in 243 and 80 accessions, respectively, in the core collection. The stratification of maize accessions by geographic region according to kernel type resulted in 21 extracts (Table 1). The extract with the highest number of accessions was Caatinga-Dent (CA-D) with 115 accessions and that with the lowest Agreste-Litoral Semiflint (AL-SF) with only one. Five extracts (S-D, CE-D, AM-SD, CA-D and CA-F) represented 50% of their respective active collection.

The core collection extract developed by using the constant sampling strategy contained 14% and 4% of C30 and C10, respectively (Table 1). The extracts S-F, S-SF, AM-SF, CA-F, AL-F and AL-SF were sampled as a whole, since they contained only a small number of accessions. The CA-D extract comprehended the highest number of accessions, with a total of 243, and the CA-D and S-D extracts a total of 80 each. Random sampling of each extract was carried out to identify accessions. The results of χ^2^ tests were significant, this strategy caused an alteration in accession frequency in each extract (Table 1).

As regards the proportional strategy, the amount of selected accessions in each extract was proportional to its size. The extract AL-SF was not sampled by P30, since it contained too few accessions (Table 1). On using the P10 strategy, the extracts S-SF, AM-SF, AL-F and AL-SF revealed an insufficient number of accessions, thus obviating their sampling. Random sampling of each extract was carried out to identify the accessions for both strategies. χ^2^ values for P30 and P10 were low, this indicating a lack of significance. Therefore, this strategy did not give rise to alterations in extract accession frequencies.

The logarithmic strategy was not applied to S-SF, AM-SF, AL-F and AL-SF extracts owing to the low number of accessions. Random sampling of each extract was carried out to identify accessions. This strategy gave χ^2^ values that indicated a lack of significance in the case of L30, whereas there was indication of significance at 1% probability for L10, due to integral sampling of S-SF, AM-SF, AL-F and AL-SF extracts, where substantial alterations in frequency could be observed.

The core collection established using the random strategy sampled 243 or 80 accessions. χ^2^ values were low, thus indicating a lack in significance (Table 1).

**Figure 1:**
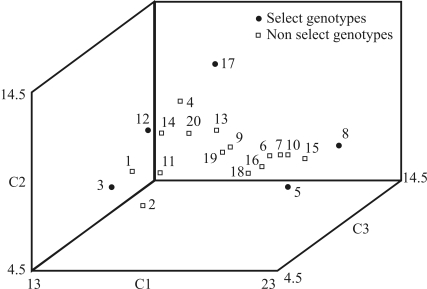
Dispersion graphic of 20 accessions of extract 3 in Janaúba based on scores in three principal components (C1, C2 and C3).

Through the multivariate strategy with 30% intensity (MV30), the number of sampled accessions was found to be proportional to the locale of evaluation. Consequently, 183 accessions were sampled in Sete Lagoas and 60 in Janaúba, makin for a total of 243 accessions. Those evaluated in Janaúba were derived mainly from the northern and northeastern regions of Brazil (Amazônia, Caatinga, Cerrados-Norte, Agreste Litoral and Cerrado). Two locale-evaluations were used to decrease the interaction of genotype x environment, and some accessions were not adapted for planting in southern Brazil. On considering stratification according to geographic region and kernel type, 13 extracts were obtained in Janaúba and 19 in Sete Lagoas (Table 2). Extracts with a reduced number of accessions were not submitted to statistical analyses, all their accessions being selected. Accessions were identified by multivariate analyses for each extract. As to extracts from Janaúba, χ^2^ tests were significant at 1% probability. This occurred due to integral sampling of extracts with a small number of accessions, thereby giving rise to sizeable alterations in accession frequency. In the case of extracts from Sete Lagoas, χ^2^ tests were not significant, although integral sampling was done with some. By means of graphs of the first three principle components (Figure 1), it was possible to observe the dispersion of accessions in each extract. However, the two most divergent genotypes, in relation to the group, may be genetically closer. This situation demonstrates the importance of graphical analysis (Figure 1). The accessions of extracts from Sete Lagoas showed a greater availability of traits when compared to those from Janaúba (Table 2). The evaluated traits of each and the same extract were not necessarily identical at each locale of evaluation.

According to the MV10 strategy, there were few accessions in the extracts CE-D, CE-SD, AM-F, AM-SF, CA-F, AL-D and AL-SD from Janaúba, and NC-D, AL-F and AL-SF from Sete Lagoas, thereby obviating submission to statistical analyses. Thus, all encountered accessions were selected (Table 3). The number of sampled accessions was proportional to the locale of evaluation, so that 20 accessions in Janaúba and 60 in Sete Lagoas were sampled, 80 all told. The χ^2^ test was significant at 1% probability, this indicating the occurrence of significant alterations in accession frequency in the extracts from Janaúba.

The variances obtained for the 18 traits of the active collection by means of the five strategies, showed few modifications in the case of L30, P30, C30 and R30 (Table 3). As to the MV30 strategy, 11 of the 18 traits revealed variances which diverged in relation to the active collection. Few alterations in averages were observed, these being significant only in the KW average in the C30 and the LNA in the L30 strategies (data not shown). The retention index was 83% for C30, 82% for P30, 85% for L30, 85% for R30 and 96% for MV30.

The retention indices were 71% for C10, 74% for P10, 79% for L10 and 89% for MV10. Only the multivariate strategy presented an index above 80%. Retention indices were greater for strategies that sampled 30% of the accessions compared to those that sampled 10%. In general, for all of the sampling strategies, a higher sampling intensity resulted in a higher retention index. These results are similar to those reported by Balfourier *et al.* (1999).

## Discussion

The stratification of a core collection based on geographic region and kernel-type has been considered adequate, with the type of kernel indicating different evolutionary origins (Brieger *et al.*, 1958), and the geographic region a different evolutionary direction (Hodgkin *et al.*, 1995). Furthermore, maize core collections based on kernel type and geographic region have already been established and evaluated in China (Li *et al,.* 2005).

Most core collections consist of between 5 and 20% of all the accessions in the total collection. In very large collections, this percentage may be lower, as is the case for the International Barley Core-Collection, which contains 1600 accessions, this representing only 0.3% of the barley base-collection (von Bothmera *et al.*, 2004). The maize core collection established in China contains 7% of the original collection, based on a previous study of sampling strategies (Li *et al.*, 2005).

The sampling of 30 and 10% of the landrace accessions resulted in 243 and 80 accessions, respectively, in the core collection. This proved to be adequate, as genetic variability was retained, and the resulting active collection consisting of 806 accessions was considered to contain a sufficient numberof accessions. There are many suggestions regarding the size of a core collection. Based on the theory of neutral alleles, and simulated scenes with different numbers and allelic frequencies for the loci of several populations, the core collection must contain at least 10% of the accessions of the total collection, if it does not contain all of the genetic variability of the species (Brown, 1989a, 1989b). However, a core collection with a maximum of 3000 accessions is permitted when the total collection contains all of the species genetic variability (Brown, 1989a). With these procedures, there is an 85% probability that the core collection will include 80% of the alleles in the whole collection. Another suggestion is that the ideal size for a core-collection is 5 to 10% of the total collection, thereby retaining 75 to 90% of genetic diversity (Bisht *et al.*, 1998). A core collection with high percentages (20%-30%) is proposed, especially when the objective is to retain the genetic diversity of quantitative traits (Noirot *et al.* ,1996). A further reason is that the ideal size for a core collection depends on genetic redundancy between accessions, the available resources for maintaining the core collection and the frequency of regenerated accessions (Yonezawa *et al.*, 1995). The sorghum core collection developed by ICRISAT (International Crops Research Institute for the Semi-Arid Tropics), although consisting of less than 3% of the base-collection, contains more than 90% of the variation therein(Yonezawa *et al.* 1995). Therefore, a perfect ratio or fixed size for all core collections does not exist, the appropriate size being specific for each case.

The results accruing from the strategies employed in the development of a core collection were different, according to the number of accessions in the extracts, although certain general conclusions can be drawn. An active collection can be characterized as medium-sized, with many extracts, some with a small number of accessions.

The constant strategy proved to be inadequate, since small rather than large groups were more represented. Furthermore, seeing that the redundancy level was higher in the latter, this strategy was detrimental to their aggregation, being indicated only when alleles rarely occured (Brown, 1989a). In short, this strategy favors small to the impairment of large groups.

As to the proportional strategy, χ^2^ values indicated a lack of significance, therey denoting the maintenance of proportionality, which per se may explain the frequent use of this strategy. However, through its intermedium, a bias that favors large groups is introduced. This is the most frequently used strategy, and has been adopted for half of the core collections (Brown and Spillane,1999). It has been cited in previous works as being more efficient than simple random sampling, since it includes more alleles and greater genetic variation (Brown and Spillane,1999).

The logarithmic strategy was inadequate, since there were many extracts with small accessions in the active collection, and also due to expressive alterations in frequency. This strategy was used with approximately 25% of the developed core collections to determine the number of entries per group (Brown and Spillane, 1999). Therefore, it is inappropriate for developing core collections of extracts with few accessions, although, it is advantageous in preventing extreme sampling of accessions from large extracts, besides increasing the number of accessions sampled in small extracts, when compared to the proportional strategy. A Chinese maize core collection was established by using logarithmic strategy to determine the number of entries in the core at each step (Li *et al.*, 2005).

Use of the random strategy did not generate significant alterations in accession frequency in each extract. Furthermore, there was greater probability of accessions from large groups being sampled, thereby guaranteeing proportionality. A core collection can be established from simple random sampling of the accessions, or by sampling accessions where the number of entries is equidistant. Nevertheless, neither strategy guarantees the formation of genetically distinct groups.

Since it was imperative to undertake statistical analyses of a multivariate strategy at the extract-level, the available information for each extract was used in the study of genetic divergence. With the exception of the S-SF extract of Sete Lagoas, the percentage of variance was above 80%, which can be explained by the first principal component (Cruz and Regazzi, 1997). Integral sampling of small groups with the MV10 strategy gave rise to marked alterations, both in the frequencies of extracts and the final result of the test. Inclusion of small groups in statistical analyses is not recommended. In the case of strategies where the sampling intensity was 30%, there was, as a result, no appreciable modification in the averages or variances of traits..

Use of the C10, P10, L10 and R10 strategies resulted in three or four modifications in trait variance in relation the active collection (Table 3). With the MV10 strategy, there were twelve traits with modifications in variance, which indicates efficient optimization of the variability. The averages of the 18 active collection traits and five accession sampling strategies were statistically similar. It can be considered that all the sampling strategies were adjusted, with retention indices above 80% (Frankel and Brown, 1984). Notably, MV30 presented the highest retention index.

Twenty four rice core collections were established by using eight hierarchical clustering methods, combined with random, preferred and deviation sampling at a sample proportion of 15% (Pkania *et al.*, 2007). These core collections were compared with others set up at sample proportions of 10% and 20%. Furthermore, the trend of increasing the sample proportion from 5% to 60% for core collection development could be achieved at a sample proportion range of 10%-25%. Further results revealed that the deviation sampling strategy in combination with the single-linkage method retained the highest degree of genetic diversity relative to the initial collection. The core collection that was developed by using a sample size of 15% retained the highest degree of diversity, remaining stable with all the clustering methods. Hence, this was the best way for developing a core collection of rice quality traits.

It is noteworthy that, independent of sampling strategy, the highest retention indices were obtained when using the multivariate analysis strategy. For this strategy, the difference between retention indices for both sampling intensities was only 7%. Therefore, in a core collection with 143 accessions there is 96% variability, and in that with 80, approximately 89%. Thus, it can be concluded that it is possible to develop a core collection with adequate representation with only 10% of the accessions.

In forming a core collection, the multivariate sampling strategy allows for a significant reduction in the size of the active collection, when considering sampling of 30% of the accessions, besides presenting the highest retention index of variability and few modifications in extract accession frequency, and averages and variance of traits.

Core collections are not static, as they are susceptible to alterations over time, so that they may vary in content and size. Therefore, following the acquisition of new information, new accessions can be introduced into the collection and old ones removed. The core-collection of peas was established with 2500 accessions and now, after 15 years of evaluation, there are only 150 (Matthews and Ambrose, 1994).

## Figures and Tables

**Table 1:** Number (NA) and frequency (F) of accessions per extract obtained from the South (S), Cerrados (CE), North Cerrados (NC), Amazônia (AM), Caatinga (CA) and Agreste-Litoral (AL) regions and the total active collection, kernel type dent (D), semident (SD), flint (F) and semiflint (SF), χ^2^ test considering the following strategies: constant (C), proportional (P), logarithmic (L), and random (R) and sampling of 10 and 30% of the accessions.

Extracts region- kernel type	Active collection			Constant			Proportional			Logarithmic			Random	
	NA	F (%)		C10 (%)	C30 (%)		P10 (%)	P30 (%)		L10 (%)	L30 (%)		R10 (%)	R30 (%)
S-D	89	11.04		6.25	5.76		11.25	11.11		0.10	8.64		11.25	11.52
S-SD	18	2.23		5.00	5.76		2.50	2.06		0.03	2.88		2.50	3.70
S-F	11	1.36		5.00	4.53		1.25	1.23		0.01	1.23		0.00	1.65
S-SF	6	0.74		5.00	2.47		0.00	0.82		61.31	2.47		0.00	0.41
CE-D	87	10.79		5.00	5.76		11.25	10.70		1.01	8.64		17.50	11.11
CE-SD	76	9.43		5.00	5.76		10.00	9.47		0.39	8.23		10.00	8.64
CE-F	42	5.21		5.00	5.76		5.00	5.35		0.01	6.17		3.75	3.70
CE-SF	23	2.85		5.00	5.76		2.50	2.88		0.28	3.70		5.00	2.47
NC-D	21	2.61		5.00	5.76		2.50	2.47		0.50	3.70		3.75	3.70
NC-SD	37	4.59		5.00	5.76		5.00	4.53		0.04	5.76		3.75	4.53
AM-D	39	4.84		5.00	5.76		5.00	4.94		0.01	5.76		2.50	4.94
AM-SD	86	10.67		5.00	5.76		11.25	10.70		0.94	8.64		12.50	8.23
AM-F	19	2.36		5.00	5.76		2.50	2.47		0.01	3.29		1.25	3.70
AM-SF	3	0.37		3.75	1.23		0.00	0.41		30.65	1.23		0.00	0.00
CA-D	115	14.27		6.25	6.17		13.75	14.40		2.13	9.47		12.50	13.17
CA-SD	81	10.05		5.00	5.76		10.00	9.88		0.65	8.23		10.00	11.93
CA-F	9	1.12		5.00	3.70		1.25	1.23		0.02	3.70		0.00	0.82
AL-D	23	2.85		5.00	5.76		2.50	2.88		0.28	3.70		1.25	3.70
AL-SD	18	2.23		5.00	5.76		2.50	2.06		0.03	2.88		2.50	1.65
AL-F	2	0.25		2.50	0.82		0.00	0.41		20.44	0.82		0.00	0.00
AL-SF	1	0.12		1.25	0.41		0.00	0.00		10.22	0.41		0.00	0.41
χ^2^	806			141^**^	62^**^		1.8^ns^	0.3 ^ns^		129^**^	19.8 ^ns^		14.0^ns^	5.8^ns^

^**^, ^ns^: Significant and not significant, respectively, by the χ^2^ test with 1% probability.

**Table 2:** Number (N) and frequence (F) of access per extract obtained from the South (S), Cerrados (CE), North Cerrados (NC) , Amazônia (AM), Caatinga (CA) and Agreste-Litoral (AL) regions and total Active Collection (AC), kernel type dent (D), semident (SD), flint (F) and semiflint (SF), with the χ^2^ test, considering multivariate strategies and sampling of 10% and 30% of access.

Janaúba						Sete Lagoas				
Extract	N (AC)	F (AC) (%)	F (MV10) (%)	F (MV30) (%)		Extract	N (AC)	F(AC) (%)	F (MV10) (%)	F (MV30) (%)
CE-D	1	0.51	5.00	1.67		S-D	89	14.64	13.33	14.75
CE-SD	3	1.52	5.00	5.00		S-SD	18	2.96	3.33	2.73
NC-D	20	10.10	5.00	8.33		S-F	11	1.81	1.67	1.64
NC-SD	37	18.69	15.00	15.00		S-SF	6	0.99	1.67	1.09
AM-D	11	5.56	5.00	5.00		CE-D	86	14.14	13.33	13.66
AM-SD	53	26.77	20.00	21.67		CE-SD	73	12.01	11.67	12.02
AM-F	2	1.01	5.00	3.33		CE-F	42	6.91	6.67	6.56
AM-SF	3	1.52	5.00	5.00		CE-SF	23	3.78	3.33	3.83
CA-D	42	21.21	15.00	18.33		CN-D	1	0.16	1.67	0.55
CA-SD	21	10.61	5.00	8.33		AM-D	28	4.61	3.33	4.37
CA-F	1	0.51	5.00	1.67		AM-SD	33	5.43	5.00	5.46
AL-D	3	1.52	5.00	5.00		AM-F	17	2.80	3.33	2.73
AL-SD	1	0.51	5.00	1.67		CA-D	73	12.01	11.67	12.02
-	-	-	-	-		CA-SD	60	9.87	8.33	9.84
-	-	-	-	-		CA-F	8	1.32	1.67	1.09
-	-	-	-	-		AL-D	20	3.29	3.33	3.28
-	-	-	-	-		AL-SD	17	2.80	3.33	2.73
-	-	-	-	-		AL-F	2	0.33	1.67	1.09
-	-	-	-	-		AL-SF	1	0.16	1.67	0.55
Total	198					Total	608			
χ2			169.7^**^	40.3^**^					34.6^ns^	3.7^ns^

**, ^ns^: Significant and not significant, respectively, by the χ^2^ test with 1% probability.

**Table 3:** Variance of 18 traits in the core-collection obtained from the constant (C), proportional (P), logarithmic (L) and random (R) strategies, and sampling of 10% and 30% of the accessions.

Traits	Active collection	C30	P30	L30	R30	MV30
DA	89.65	114.91^**^	88.27^ns^	93.75^ns^	95.31^ns^	109.34^ns^
DS	103.37	133.19^**^	100.66^ns^	108.74^ns^	106.95^ns^	128.94^ns^
PH	1655.16	1577.70^ns^	1559.53^ns^	1727.40^ns^	1763.72^ns^	2062.78^ns^
EH	1082.06	1051.21^ns^	1008.65^ns^	1084.14^ns^	1109.79^ns^	1304.18^ns^
PBP	178.63	177.2^**^	168.54^**^	237.01^ns^	160.38^**^	266.64^ns^
PLP	169.84	108.5^**^	134.25^**^	179.08^ns^	218.41^**^	265.15^**^
LNA	1.13	0.95^ns^	1.22^ns^	4.00^**^	1.29^ns^	2.48^**^
LN	2.82	2.51^ns^	3.14^ns^	2.28^ns^	2.78^ns^	3.77^**^
SW	7.28	7.65^ns^	6.48^ns^	6.61^ns^	8.23^ns^	9.30^ns^
NE	0.02	0.02^ns^	0.02^ns^	0.02^ns^	0.02^ns^	0.03^**^
EL	320.21	378.91^ns^	314.76^ns^	347.91^ns^	350.69^ns^	531.51^**^
EW	17.28	16.24^ns^	16.68^ns^	21.13^ns^	18.99^ns^	27.36^**^
NRK	3.65	4.59^ns^	3.75^ns^	4.09^ns^	3.69^ns^	6.36^**^
NKR	23.82	24.62^ns^	25.13^ns^	23.46^ns^	24.97^ns^	38.04^**^
EW	1132.02	1118.90^ns^	1173.33^ns^	1177.14^ns^	1280.56^ns^	1994.39^**^
GW	840.41	828.59^ns^	883.00^ns^	922.62^ns^	919.76^ns^	1493.15^**^
CW	6.53	7.26^ns^	6.15^ns^	6.62^ns^	6.52^ns^	8.96^**^
KW	2891.35	2826.07^ns^	3085.18^ns^	3078.49^ns^	2639.89^ns^	3643.0^ns^

		C10	P10	L10	R10	MV10

DA	89.65	117.06^ns^	75.29^ns^	78.46^ns^	94.70^ns^	142.63^**^
DS	103.37	130.43^ns^	102.82^ns^	85.20^ns^	102.82^ns^	174.02^**^
PH	1655.16	2096.16^ns^	1154.20^ns^	2058.44^ns^	1286.39^ns^	2243.15^ns^
EH	1082.06	1292.51^ns^	960.04^ns^	1197.45^ns^	948.08^ns^	1523.41^ns^
PBP	178.63	147.54^**^	338.19^ns^	186.01^ns^	273.46^ns^	196.92^ns^
PLP	169.84	64.06^**^	149.95^ns^	149.39^ns^	182.35^ns^	212.59^ns^
LNA	1.13	1.51^ns^	2.42^**^	4.32^**^	0.30^**^	5.06^**^
LN	2.82	3.06^ns^	4.11^ns^	28.21^**^	2.34^ns^	4.55^**^
SW	7.28	5.76^ns^	5.93^ns^	69.68^**^	7.10^ns^	10.19^ns^
NE	0.02	0.02^ns^	0.03^**^	0.16^**^	0.01**	0.04^**^
EL	320.21	275.41^ns^	356.65^ns^	364.73^ns^	379.63^ns^	673.80^**^
EW	17.28	16.37^ns^	20.96^ns^	18.35^ns^	19.59^ns^	28.12^**^
NRK	3.65	2.36^**^	6.80^**^	2.49^ns^	6.78^**^	8.96^**^
NKR	23.82	21.19^ns^	23.97^ns^	24.65^ns^	25.66^ns^	50.10^**^
EW	1132.02	1242.96^ns^	1274.42^ns^	1463.0^ns^	1328.1^ns^	2617.0^**^
GW	840.41	951.39^ns^	887.40^ns^	979.28^ns^	1067.70^ns^	1977.2^**^
CW	6.53	5.67^ns^	8.16^ns^	6.76^ns^	7.71^ns^	10.45^**^
KW	2891.35	2912.99^ns^	3517.50^ns^	2333.57^ns^	4149.47^ns^	3978.39^ns^

^**^, ^ns^: Significant and not significant, respectively, by the χ^2^ test with 1% probability. Days to anthesis (DA); Days to silking (DS); Plant Height (PH); Ear Height (EH); Percentage of broken plants (PBP); Percentage of lodging plants (PLP); Leaf number above the superior ear (LNA); Leaf number per plant (LN); Stalk width (SW); Number of ears (NE); Ear Length (EL); Ear width (EW); Number of rows of grains (NRG); Number of grains per row (NGR); Ear Weight (EW); Grain Weight per plant (GW); Cob Weight (CW); 1000 kernels Weight (KW).
